# Development of an alternative saliva test for diagnosis of SARS-CoV-2 using TRIzol: Adapting to countries with lower incomes looking for a large-scale detection program

**DOI:** 10.1371/journal.pone.0255807

**Published:** 2021-08-18

**Authors:** Haydee Miranda-Ortiz, Edith A. Fernández-Figueroa, Erika B. Ruíz-García, Anallely Muñoz-Rivas, Alejandra Méndez-Pérez, Jorge Méndez-Galván, Horacio Astudillo-de la Vega, Belem Gabiño-López, Ricardo Nava-Monroy, Alejandro López-Flores a la Torre, Tanit López-Vergara Anaya, Diana Vilar-Compte, Uriel Coquis-Navarrete, Melissa Valdés-Reyes, Sokani Sánchez-Montes, Ingeborg Becker

**Affiliations:** 1 Sequencing Unit, National Institute of Genomic Medicine, Mexico City, Mexico; 2 Computational and Integrative Genomics, National Institute of Genomic Medicine, Mexico City, Mexico; 3 Centro de Medicina Tropical, División de Investigación, Facultad de Medicina, Universidad Nacional Autónoma de México, Mexico City, Mexico; 4 Department of Gastrointestinal Medical Oncology, Instituto Nacional de Cancerología, Mexico City, Mexico; 5 Translational Medicine Laboratory, Instituto Nacional de Cancerología, Mexico City, Mexico; 6 Genomic Diagnostic Laboratory, National Institute of Genomic Medicine, Mexico City, Mexico; 7 Clínica Sansce, Mexico City, Mexico; 8 Dirección de Investigación, Hospital Infantil de México “Federico Gómez”, Mexico City, Mexico; 9 Translational Research Laboratory in Cancer & Cellular Therapy, Hospital de Oncologia, Siglo XXI, IMSS, Mexico City, Mexico; 10 Laboratory of Histology and Confocal Microscopy, National Institute of Genomic Medicine, Mexico City, Mexico; 11 Department of Infectious Diseases, Instituto Nacional de Cancerología, Mexico City, Mexico; 12 Facultad de Ciencias Biológicas y Agropecuarias región Tuxpan, Universidad Veracruzana, Veracruz, Mexico; "INSERM", FRANCE

## Abstract

The use of saliva for the diagnosis of SARS-CoV-2 has shown to be a good alternative to nasopharyngeal swabs (NPS), since it permits self-collection, avoids the exposure of healthy persons to infected patients, reduces waiting times, eliminates the need of personal protective equipment and is non-invasive. Yet current saliva testing is still expensive due to the need of specialized tubes containing buffers to stabilize the RNA of SARS-CoV-2 and inactivate the virus. These tubes are expensive and not always accessible in sufficient quantities. We now developed an alternative saliva testing method, using TRIzol for extraction, viral inactivation, and storage of SARS-CoV-2 RNA, combined with RT-qPCR, which was comparable in its performance to NPS. Paired saliva samples and NPS were taken from 15 asymptomatic healthcare workers and one patient with SARS-CoV-2. Further 13 patients with SARS-CoV-2 were only saliva-tested. All the tests were performed according to CDC 2019-Novel Coronavirus (2019-nCoV) Real-Time RT-PCR Diagnostic Panel. Saliva (4 mL) was taken in sterile 50 mL tubes, 1.5 mL TRIzol were added and mixed. Our results show that 5 μL of saliva RNA extracted with TRIzol allow for an adequate detection of the virus in patients positive for SARS-CoV-2 and was equally sensitive to NPS in TRIzol. We conclude that saliva testing using TRIzol is a recommendable method for diagnosis of SARS-CoV-2 since it has several advantages over currently used saliva tests: it can be done with normal sterile tubes, does not need cold-chain handling, is stable at room temperature, is non-invasive and less costly, making it more accessible for low-income countries. Cheaper saliva testing using TRIzol is especially relevant for low-income countries to optimize diagnosis and help define quarantine durations for families, healthcare workers, schools, and other public workplaces, thus decreasing infections and mortality caused by SARS-CoV-2.

## Introduction

The increasing spread of SARS-CoV-2 calls for rapid, accurate and easy to handle diagnostic tests, which are essential for controlling the ongoing pandemic. Although the current golden standard consists of nasopharyngeal swabs (NPS) to be analyzed by quantitative reverse-transcriptase polymerase chain reaction (RT-qPCR), an alternative diagnostic sampling method using salivary fluid is becoming more widely used to diagnose SARS-CoV-2 [1–3]. Salivary testing has proven to have important advantages over NPS: it permits self-collection, reducing handling by healthcare workers and thereby avoiding biosafety risks of coming in contact with aerosol or droplets. Furthermore, it reduces the exposure of healthy persons to the proximity of infected patients, while waiting to be tested in hospitals or laboratories. It reduces waiting times, it lowers costs by eliminating the need of personal protective equipment and, above all, it is a non-invasive procedure.

SARS-CoV-2 RNA is detectable in the oropharyngeal cavity; however, respiratory sample collection can cause discomfort to the patients. Therefore, saliva is an excellent diagnostic fluid sample and had been used for detection of SARS-CoV-2 through methods such as RT-qPCR, viral culture, RT-PCR, RT-LAMP (reverse transcription loop-mediated isothermal amplification) and antigen test [4, 5].

Saliva is a hypotonic fluid consisting of 99% water, containing multiple electrolytes, enzymes, immunoglobulins, and antimicrobial factors. It represents an attractive biofluid for the detection of diverse viral infections, including Epstein Barr virus, Human Papillomavirus, HIV, as well as respiratory viruses such as influenza, parainfluenza virus and respiratory syncytial virus [6]. More recently, SARS-CoV-2 has also been detected in saliva [7, 8]. The entry of SARS-CoV-2 to saliva can occur through diverse routes: blood circulation, extracellular vesicles secretion by infected cells, droplets from the respiratory tract, gingival crevicular fluid and by infection of the oral mucosal lining [9].

To date, most diagnostic strategies for COVID-19 detection in saliva require specialized tubes containing buffers that stabilize the RNA of SARS-CoV-2 and inactivate the virus. Yet these tubes are expensive and not always accessible in sufficient quantities. Thus, despite that saliva is a promising type of sample that can be self-collected for the diagnosis of SARS-CoV-2, the cost and availability of specific collection tubes limit widespread testing efforts.

Therefore, the development of more affordable and easier collection options is needed due to the rising demands of testing, not only for patients but also for the continued surveillance of health care workers and front-line professionals. We here report the development a saliva test using TRIzol that can be done by self-collecting, does not require special tubes or inactivation buffer, yet has all the above-mentioned advantages, including easy and safe handling, does not require cold storage, remains stable for a week at room temperature, is highly sensible, non-invasive, and less costly.

## Materials and methods

The study was approved by the Ethics and Research Committee of the Faculty of Medicine, UNAM with Approval No. FM/DI/026/2020, and guidelines established by the Mexican Health Authorities were strictly followed. Additionally, an official written informed consent was signed by the patients, as well as by the tested health care workers, accepting the qPCR-SARS CoV-2 diagnosis. The patients (aged between 22 and 85 years) also signed a written informed consent allowing the use of their samples for research purposes, once they had received their results.

### Samples of patients and health care workers

Paired saliva samples and NPS were taken between October and November 2020 from 15 asymptomatic healthcare workers at the Tropical Medical Center of the Faculty of Medicine, UNAM, Clinica Sansce and the National Cancer Institute in Mexico, to screen for SARS-CoV-2. Both types of samples (saliva and NPS) were also taken from a patient who had tested positive for SARS-CoV-2. Additionally, saliva was tested in 13 patients with SARS-CoV2, according to the criteria established by CDC 2019-Novel Coronavirus (2019-nCoV) Real-Time RT-PCR Diagnostic Panel.

All the NPS were taken by a trained nurse using 2 cotton swabs (one for the oropharyngeal- and one for the nasopharyngeal swabs) (Cleanmo), which were introduced into a sterile tube containing 1 mL TRIzol reagent (Invitrogen or Biosciences). In case of saliva sampling, the patients received a sealable plastic bag containing a 50 mL tube for saliva, a 2 mL tube with 1.5 mL of TRIzol, a paper towel and two gloves. The patient was asked to expel 4 mL saliva into the sterile 50 mL tube (on an empty stomach, without having performed oral hygiene) and thereafter to add 1.5 mL TRIzol to the tube containing the saliva, mix it and seal the bag (containing the 50 ml tube with saliva with TRIzol, the paper towel, the empty 2 mL tube and the gloves). A nurse received the sealed bag wearing gloves and deposited it into a cooler containing freezer packs for transport to the laboratory. This could be done at the patient’s home.

### RNA extraction

Upon arrival in the laboratory, the samples containing the 4 mL saliva and 1.5 mL TRIzol were homogenized for 2 min in a vortex, incubated during 10 min at RT and kept at 4°C (6–24 hours) until RNA extraction was done. One mL of the saliva/TRIzol solution was taken and mixed with 200 μL cold chloroform (Sigma). This solution was incubated during 5 min and centrifuged at 19,357 x g for 10 min at RT. The aqueous phase (500–600 μL) was recovered and 700 μL isopropanol at RT (Sigma) were added. The resulting solution was mixed for 15 s and incubated during 10 min (when extraction was done the same day) or overnight at -20°C (if extraction was finished the next day). Thereafter, the solution was centrifuged at 19,357 x g for 10 min at RT. The supernatant was discarded and 1 mL ethanol 80% (Sigma) was added, the solution was mixed during 10 s and centrifuged at 19,357 x g during 10 min at RT. The ethanol was discarded, the excess was dried at RT and the pellet was suspended in 50 μL RNAse free water and stored at -20°C until use.

For the comparison of saliva extraction vs swabs, two swabs were taken and incubated during one hour in 1 mL of TRIzol, and the RNA extraction was done as described before.

Tests were done with RNA extracted from saliva and NPS.

### Retro-transcription and qPCR

Total RNA (5 μL) was analyzed for the detection of SARS-CoV-2 using N1 and N2 probes (nucleocapsid protein gene) and RNAse P human gene (Cat. 10006770. 2019-nCov CDC EUA Kit, IDT), using GoTaq Probe 1-Step RT-qPCR System (Cat. A6121, Promega) in a reaction volume of 20 μL containing: 10 μL GoTaq, 1.5 μL probe (N1, N2 or RNAse P), 0.4 μL GoScript, 3.1 μL Nuclease-Free water and 5 μL RNA. As positive control, Synthetic DNA Gene N of SARS-CoV-2 (Cat. 10006625. 2019-nCoV_N_ Positive Control, IDT) was used. The thermal profile was as follows: 45°C during 15 min, 95°C during 2 min, 40 cycles at 95°C for 15 s and 60°C for 1 min. The 7500 FAST Real-Time PCR System was used.

Ct values were assessed by qRT-PCR described in the CDC 2019 Novel Coronavirus (2019-nCoV) RT-PCR Diagnostic Panel instructions (CDC-006-00019).

### Linearity assay

For the linearity assays, duplicates of standard curves were produced from serial dilutions, ranging from 80,000 to 78 copies/ μL, using Synthetic DNA of the N gene of SARS-CoV-2 (Cat. 10006625). As positive control, 2019-nCoV_N_Positive Control (IDT) was used in a 1:4 dilution. The qPCR assays were performed as described.

### Statistical analysis

Statistically significant changes between groups were assessed using the one sample t, paired t and Mann-Whitney U tests. A value of p≤0.05 was considered significant. The analysis was done using the Prism 9 software (GraphPad Software, San Diego, CA, USA).

## Results

### Linearity assay

**Fig 1:**
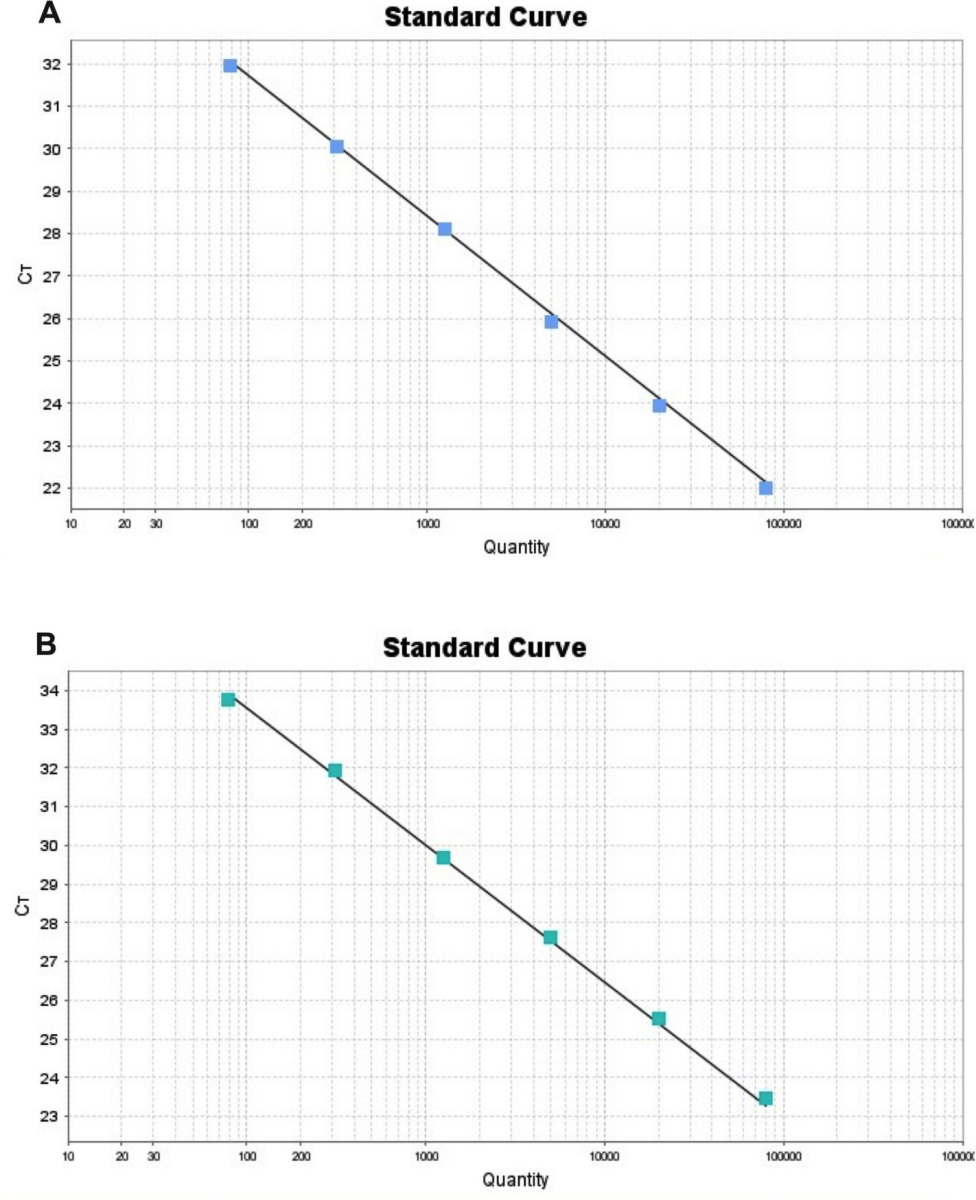
Dynamic extension of the SARS-CoV-2 N gene. (A) Standard curve for the target N1. (B) Standard curve for the target N2. Serial dilutions of synthetic DNA of the N gene were assayed in a 1:4 proportion for both curves.

Linearity assay was performed to determine the dynamic extension for the control N gene of SARS-CoV-2. A dynamic extension of six points in a proportion 1:4 by duplicate ranging from 80,000 to 78.12 copies/ μL was obtained (Fig 1A and 1B and Table 1) with an R^2^ = 0.994, a slope of -3.28 and a percentage of efficiency of 101.3 for N1. For N2, an R^2^ = 0.998, a slope of -3.54 and a percentage of efficiency of 91.5 was obtained. The qPCR assay was performed in monoplex with synthetic DNA of the N gene from SARS-CoV-2. Linearity of the 6 points was found in duplicate studies.

**Table 1:** Linearity assay of the N gene of SARS-CoV-2.

Quantity (copies/uL)	Ct N1	Ct mean	Ct SD	STD curve results
80,000	21.99	22.05	0.07	Slope: -3.28 R^2^: 0.994 Eff%: 101.3
20,000	23.94	23.99	0.06	
5,000	25.93	26.43	0.7	
1,250	28.10	28.11	0.01	
312.5	30.05	30.04	0.01	
78.12	31.94	31.94	0.002	
Quantity (copies/uL)	Ct N2	Ct mean	Ct SD	STD curve results
80,000	23.47	23.17	0.42	Slope: -3.54 R2: 0.998 Eff%: 91.5
20,000	25.51	25.48	0.04	
5,000	27.63	27.56	0.09	
1,250	29.68	29.63	0.06	
312.5	31.94	31.87	0.09	
78.12	33.77	33.85	0.12	

### Detection of SARS-CoV-2 N1 and N2 (nucleocapsid targets) in saliva samples and NPS

An assay comparing Ct values of paired saliva and NPS (n = 15) of healthy asymptomatic individuals (healthcare workers) was conducted, using the human gene coding for the RNA subunit of ribonuclease P (RNAse P). As internal control, the 2019-nCov CD Kit was included, which confirmed the presence of viral RNA in the extracted samples (Table 2). Our results show that generally the Ct values of RNAse P in saliva were higher (with an average of 2.48), as compared to the Ct of NPS. A statistical analysis of the CT values comparing NSP and saliva tests was made using a paired t test (Table 2). A significative difference was found between the samples (p value = 0.0037). It is noteworthy, that both samples complied with Ct values (≤35) required for quality control, according to CDC guidelines for the diagnosis of SARS CoV-2.

**Table 2:** Comparison of RNAse P in paired samples (NPS and saliva) of healthcare workers (HW).

ID sample	Ct RNAse P	Ct RNAse P Saliva
	NPS	
HW 1	26.0	28.5
HW 2	25.0	27.9
HW 3	24.2	28.2
HW 4	26.0	32.4
HW 5	29.0	24.9
HW 6	26.1	29.5
HW 7	25.8	28.9
HW 8	24.1	27.6
HW 9	23.2	27.9
HW 10	23.3	25.3
HW 11	23.0	24.2
HW 12	29.5	30.4
HW 13	23.7	27.3
HW 14	24.0	29.4
HW 15	25.8	23.4

We also performed a one sample t test to determine whether the median of the sample is equal to a known standard value, which in our case was the Ct value (35) established in the CDC protocol. Both tests, one for NPS and one for saliva, were statistically significant (p value = 0.0001), with a mean of 25.25 and 27.72 for NPS and saliva, respectively. With these data we showed that saliva and NPS extracted with TRIzol can be used for the diagnosis of SARS-CoV-2.

In addition to healthy subjects, RNA was extracted from paired samples (NPS and saliva) of a patient who had tested positive for SARS-CoV-2 (Table 3).

**Table 3:** Comparison of RNAse P, N1 and N2 Cts in a positive patient with SARS-CoV2, using paired samples (NPS and saliva).

ID sample	Ct RNAse P	Ct N1	Ct N2
NPS	24.1	22.4	23.4
Saliva	27.6	26	27.5

Even though the Ct values were higher in saliva, as compared to NPS, SARS-CoV-2 detection was positive in both samples.

Finally, we analyzed saliva of 13 patients who had tested positive for SARS-CoV-2. All the patients complied with the criteria established by CDC 2019-Novel Coronavirus (2019-nCoV) Real-Time RT-PCR Diagnostic Panel. All 13 patients showed adequate Ct values for RNAse P, N1 and N2 targets, allowing for the detection of SARS-CoV-2 in saliva. Thus, 5 μL of saliva RNA allow for an adequate detection of the virus in patients positive for SARS-CoV-2 (Table 4).

**Table 4:** RNAse P, N1 and N2 Cts in saliva samples of patients with SARS-CoV-2.

Sample ID	Ct RNAse P	Ct N1	Ct N2
Patient 1	25.0	30.7	31.2
Patient 2	23.0	24.5	25.1
Patient 3	24.0	34.4	35.3
Patient 4	35.2	28.3	30.7
Patient 5	27.6	26.0	27.5
Patient 6	24.2	35.5	36.3
Patient 7	28.2	27.3	28.5
Patient 8	25.5	31.2	31.2
Patient 9	26.6	35.6	33.9
Patient 10	26.0	36.9	37.2
Patient 11	29.7	31.5	30.7
Patient 12	23.9	23.3	25.4
Patient 13	24.6	32.9	33.0

### Stability of samples at room temperature

Finally, an assay comparing Ct values of saliva of seven individuals, including one positive patient infected with SARS CoV-2 and 6 negative controls, was performed during 14 days at room temperature. Saliva was collected with TRIzol, as previously described. The samples were mixed and stored at 20–25°C during 1, 3, 7 and 14 days. On each of these days, RNA extraction and qPCR were done. The Cts showed similar results throughout the different days (Table 5). The statistical analysis of the data using RNAse P Cts was done using the Mann Whitney test, showing no significant differences. This confirms that saliva samples diluted in TRIzol and stored at room temperature can be used throughout 14 days, without altering the diagnostic results.

**Table 5:** Results of qPCR on days 1, 4, 7 and 14 after saliva samples were stored at room temperature (20-25° C).

Day after saliva collection.	Probe	Person 1	Person 2	Person 3	Person 4	Person 5	Person 6	Person 7
1	N1	19	Undet	Undet	Undet	Undet	Undet	Undet
1	N2	24	Undet	Undet	Undet	Undet	Undet	Undet
1	RP	29.3	24.7	32.7	25.9	27	27.4	29
3	N1	22.2	Undet	Undet	Undet	Undet	Undet	Undet
3	N2	23.9	Undet	Undet	Undet	Undet	Undet	Undet
3	RP	28.6	24.6	31.2	25.9	27.5	27.4	28.7
7	N1	23.5	Undet	Undet	Undet	Undet	Undet	Undet
7	N2	24.8	Undet	Undet	Undet	Undet	Undet	Undet
7	RP	28.8	24.4	31.5	26.3	28	26	28.4
14	N1	22.7	Undet	Undet	IS	Undet	Undet	IS
14	N2	24.9	Undet	Undet	IS	Undet	Undet	IS
14	RP	28.5	23.9	32	IS	26.3	25.6	IS

Undet: not amplified. IS: insufficient sample to perform the extraction.

Finally, the costs of different RNA extraction methods were analyzed. Important differences were found between both types of methods (TRIzol vs commercial kits), showing that saliva or NPS testing using TRIzol is less costly than to place the samples in transport medium and to use commercial kits (S1 Table).

## Discussion

Saliva is secreted by diverse salivary glands distributed throughout the oral cavity (parotid, sublingual, submandibular, minor salivary glands). They are very permeable, allowing for easy passage and exchange of diverse molecules, including viral nucleic acids, from blood capillaries surrounding the glands, to saliva [10]. Epithelial cells of the oral mucosa and those lining the minor salivary glands ducts express angiotensin II-converting enzyme receptor (ACE2), a SARS-CoV-2 receptor [11, 12]. Therefore, they may become host cells for the virus and lead to inflammatory reactions, releasing SARS-CoV-2 viral particles into the saliva. This has already been shown in Rhesus macaque primates [13, 14]. High viral loads are often found in the morning during 5–7 days after onset of symptoms, in nasopharynx, pharynx and saliva [1, 15].

Home saliva collection for diagnosis of SARS-CoV-2 was first approved by FDA on May 7, 2020 [16] and has become a promising alternative to NPS, since the latter can cause pain, discomfort and, due to the vascularity of the nasopharyngeal mucosa, swabs can rarely lead to epistaxis if not taken properly. Saliva testing has the additional advantage that it can be sent to families who can gather their saliva in sterile tubes, without leaving their homes. This is specifically convenient for service employees who need to be tested regularly [17, 18].

It is noteworthy, that saliva tests or NPS were equally sensitive in detecting SARS-CoV-2 for diagnostic purposes [19]. A further support for using saliva for detection of SARS-CoV-2 is that both viral RNA, as well as the virus, conserves their stability in saliva without supplementation. SARS-CoV-2 RNA is not degraded and remains stable even at warm temperatures, suitable for nucleases [20]. RNA samples of patients infected with SARS-CoV-2 have shown to be stable at 4°C, or room temperature for prolonged periods of time (weeks). This contrasts with human RNA, which degrades in the absence of stabilization buffers [21]. Yet, if human saliva RNA is placed in TRIzol, it can be stored at –80°C and remains stable for more than two years without the need of RNAse inhibitors [22].

TRIzol is a reagent used for extraction of RNA (in addition to DNA or proteins) from a variety of samples. It prevents degradation of RNA, preserving both its quality and integrity during the purification process and additionally permits to obtain large quantities. When compared to other extraction methods, TRIzol has been shown to maintain the quality standards of RNA [23]. This reagent is not only used for extraction, but also serves well as a storage medium. Taking into consideration that RNA of SARS-CoV-2 does not require specialized buffers for RNA stabilization and is stable at various temperatures [21], the use of TRIzol for extraction of SARS-CoV-2 RNA is promising, can decrease testing costs, inactivate the virus, and obtain stable RNA without the need of cold handling. This is particularly important for limited resources scenarios, where massive testing is required despite economic shortages.

An additional advantage of the saliva testing over NSP testing becomes evident in cases of samples where RNAse P Ct values are higher than 35. In these cases, CDC guidelines for quality control establish that the sample needs to be discarded and replaced by a new one taken from the patient. In the case of NPS testing, this would imply the need for a new test, since these samples can only be used for one test. In contrast, in the case of saliva testing, no additional samples would need to be taken, since a new extraction and RT-qPCR could be done with the remaining saliva (each patient gives 4 mL of saliva, which is stored until definite results are established). Thus, the use of saliva avoids having to re-test the patient.

Our data now show that extraction efficiency of SARS-CoV-2 RNA with TRIzol from saliva was very similar to that obtained by NPS with comparable Ct values. Our data are consistent with Wyllie et al., who showed that the same primer sequences that we used, detected more SARS-CoV-2 RNA copies in the saliva specimens than in the NPS, suggesting that both sample types had similar sensitivity for the detection of the virus [19]. This not only supports the use of TRIzol for RNA extraction, but also of using saliva instead of NPS for the diagnosis of SARS-CoV-2. Furthermore, the collection of saliva samples with TRIzol can be easily standardized using a constant volume of saliva and TRIzol and its handling can easily be explained to the patients.

Yet saliva tests can have some drawbacks that need to be considered. In some sick or dehydrated patients, saliva processing can be difficult, due thick saliva that can be stringy and difficult to pipet [24]. Furthermore, special care should be taken when it comes to child specimens, since a lower sensitivity has been reported [25, 26].

Despite the later disadvantages, saliva testing poses an important advantage, permitting self-surveillance studies, the detection of asymptomatic persons or pre-symptomatic patients without any discomfort, allowing wider testing of people [19]. Furthermore, saliva sampling by patients themselves diminishes the risk of nosocomial transmission between infected patients and health care workers and alleviates demands for supplies of swabs and personal protective equipment, lowering the costs of the diagnosis [19, 27]. Another important advantage of using saliva for obtaining SARS-CoV-2 RNA is the feasibility of being able to collect larger sample volumes as compared to NPS, which permits to easily repeat RNA extractions in case of technical errors, or to do sequence analysis without having to re-sample the patient. Furthermore, larger sample volumes potentially permit to generate BioBanks for future research, including the sequencing of the virus for the detection of mutations, the detection and quantification of antibodies, the identification of other associated pathogens and the detection of new biomarkers associated with the disease and the immune response [27].

## Conclusions

More flexible options for screening for SARS-CoV-2 RNA, such as saliva sampling, could be a useful cost-effective alternative for large-scale detection and monitoring of SARS-CoV-2. We now report a sensitive and accurate salivary diagnostic tool for self-testing, using TRIzol for RNA extraction and storage, that does not require cold-chain handling, remains stable for long periods, which not only stabilizes SARS-CoV-2 RNA but also inactivates the virus, permitting easy and safe transport and handling. The scientific evidence suggests that saliva testing using TRIzol could present a compelling alternative to other SARS-CoV-2 sampling tests, which not necessarily requires trained personnel to collect a sample, does not need the use of valuable personal protective gear, nor puts health-care professionals at risk and is more feasible for low-income countries.

Cheaper saliva testing using TRIzol is especially relevant for low-income countries to optimize diagnosis and help define quarantine durations for families, healthcare workers, schools, and other public workplaces, thus decreasing infections and mortality caused by SARS-CoV-2.

## Supporting information

S1 Table Comparison of financial costs of saliva or NPS testing with TRIzol and those employing transport medium or commercial kits, for diagnosis of SARS-CoV-2. (XLSX)
